# Neutrophil-to-Lymphocyte and Platelet-to-Lymphocyte Ratios as Predictors of Outcomes in Patients With Unresectable Hepatocellular Carcinoma Undergoing Transarterial Chemoembolization Plus Sorafenib

**DOI:** 10.3389/fmolb.2021.624366

**Published:** 2021-05-28

**Authors:** Lei Zhang, Zhi-Ping Yan, Zhong-Heng Hou, Peng Huang, Min-Jie Yang, Shuai Zhang, Shen Zhang, Shao-Hua Zhang, Xiao-Li Zhu, Cai-Fang Ni, Qiang Li

**Affiliations:** ^1^ Department of Interventional Radiology, The First Affiliated Hospital of Soochow University, Suzhou, China; ^2^ Department of Interventional Radiology, Zhongshan Hospital, Fudan University, Shanghai, China; ^3^ Shanghai Institution of Medical Imaging, Shanghai, China; ^4^ National Clinical Research Center for Interventional Medicine, Shanghai, China; ^5^ Institute of Urology, The Affiliated Luohu Hospital of Shenzhen University, Shenzhen, China; ^6^ Department of Radiology, The Affiliated People’s Hospital of Ningbo University, Ningbo, China

**Keywords:** transarterial chemoembolization, sorafenib, platelet count, neutrophils, lymphocytes

## Abstract

**Objectives:** To investigate the predictive value of inflammatory biomarkers in patients with unresectable hepatocellular carcinoma (HCC) for outcomes following the combination treatment of transarterial chemoembolization (TACE) plus sorafenib.

**Materials and Methods:** A total of 314 (270 male and 44 female) treatment-naïve patients with unresectable HCC treated by TACE plus sorafenib between January 2011 and December 2018 were enrolled in the retrospective study. The primary outcome was overall survival (OS). The secondary outcome was progression-free survival (PFS). Neutrophil-to-lymphocyte ratio (NLR) and platelet-to-lymphocyte ratio (PLR) were obtained within 3–7 days before the initial TACE and the median value of the NLR and PLR was considered as the cut-off value.

**Results:** The median value of NLR and PLR was 2.42 and 100, respectively. The median OS and PFS of the entire cohort were 18.7 months (95% CI: 16.8–20.6) and 9.1 months (95% CI: 8.5–9.8), respectively. The low NLR and PLR group showed improved OS and PFS compared with the high NLR and PLR group [21.8 months (95% CI: 15.2–28.5) vs. 15.4 months (95% CI: 12.4–18.3), *p* < 0.0001; 21.6 months (95% CI: 15.8–27.5) vs. 14.9 months (95% CI: 11.9–17.8), *p* = 0.00027, respectively]. In addition, the low NLR and PLR group also provided a longer PFS than the high NLR and PLR group [10.4 months (95% CI: 8.9–12.0) vs. 8.1 months (95% CI: 7.1–9.2), *p* = 0.00022; 10.3 months (95% CI: 8.6–11.9) vs. 8.2 months (95% CI: 7.2–9.2), *p* < 0.0001, respectively]. High NLR and PLR at baseline were predictive factors of poor OS (*p* = 0.02 and *p* = 0.004) and PFS (*p* = 0.045 and *p* = 0.005).

**Conclusion:** This study showed the prognostic value of quantitative inflammatory biomarkers in correlation with OS and PFS in unresectable HCC patients undergoing TACE plus sorafenib treatment.

## Introduction

Hepatocellular carcinoma (HCC) is the most common primary hepatic tumor and the third leading cause of cancer-related death worldwide (Forner et al., 2018; Kim and Shin, 2019). Transarterial chemoembolization (TACE) is the most widely applied treatment regimen not only in intermediate stage HCC recommended by guidelines but in advanced stage according to the BRIDGE study (Park et al., 2015; European Association for the Study of the Liver, 2018). However, the TACE-induced liver function deterioration and upregulation of vascular endothelial growth factor (VEGF) may lead to tumor recurrence and metastasis, which, in turn, provides unfavorable outcomes in unresectable HCC patients (Wang et al., 2008).

As an oral multikinase inhibitor, sorafenib has been shown to significantly offer clinical benefits in HCC patients with advanced stage (Cheng et al., 2009). Considering that sorafenib suppresses the surge of proangiogenic factors after the administration of TACE, the combination of TACE and sorafenib could have a synergistic effect and improve clinical prognosis in unresectable HCC patients (Pawlik et al., 2011). Although three trials conducted previously (post-TACE trial, SPACE trail, and TACE-2 trial) comparing clinical benefits in the combination therapy with that in TACE monotherapy did not demonstrate any compelling evidence by the addition of sorafenib to TACE, the TACTIS trial indicated that much longer duration of sorafenib administration which may prolong the progression-free survival (PFS) and provide the preservation of the liver function, which could eventually lead to prolongation of overall survival (OS) (Kudo et al., 2011; Lencioni et al., 2016; Meyer et al., 2017; Kudo et al., 2020). However, the high heterogeneity of unresectable HCC may result in heterogeneous therapeutic efficacy in patients treated by the combination of TACE plus sorafenib (Kudo, 2018). It is difficult to predict prognosis in individual patients. Therefore, development of effective biomarkers to identify subpopulations of patients who are most likely to benefit from such a combination therapy is needed (Zhang et al., 2020b). Previous studies showed that sorafenib-related adverse events were associated with favorable prognosis in HCC patients (ref.). Nevertheless, given the high treatment costs, drug-related toxicity, and the harm of ineffective treatment, cheap and readily available predictive biomarkers are of interest.

Concerning the inflammatory and immune environments that contribute to HCC formation and progression, the peripheral blood cells have been reported as a biomarker because neutrophils and high platelets suppress antitumoral immune cell function and induce upregulation of vascular endothelial growth (Zheng et al., 2017a; Schobert et al., 2020). Previous studies showed that high neutrophil-to-lymphocyte (NLR) and platelet-to-lymphocyte (PLR) values are associated with worse OS, which could have the potential to serve as quantitative biomarkers for individual prognosis prediction of HCC patients treated with TACE or sorafenib alone (Chon et al., 2019; He et al., 2019; Hong et al., 2019; Wang et al., 2020). Nevertheless, the same prognostic value of the biomarkers for unresectable HCC patients undergoing the combination therapy of TACE and sorafenib is not well studied. Consequently, the aim of this study was to investigate the effectiveness of the prognostic roles of NLR and PLR in unresectable HCC patients receiving TACE plus sorafenib treatment.

## Materials and Methods

### Patients

This retrospective study was approved by the Institutional Review Board (IRB) at two institutions. All clinical practices and observations were conducted in accordance with the Declaration of Helsinki. The requirement to obtain informed consent was waived due to the retrospective nature. The dual-center (The First Affiliated Hospital of Soochow University and Zhongshan Hospital) study consisted of consecutive treatment-naïve unresectable HCC patients treated with the combination therapy of TACE and sorafenib between January 2011 and December 2018. HCC was diagnosed by pathologic assessment or noninvasive criteria according to the European Association for the Study of the Liver (EASL) guidelines (European Association for the Study of the Liver, 2018). Each treatment decision was assessed by a multidisciplinary consensus, including interventional radiologists, oncologists, liver surgeons, and hepatologists.

The inclusion criteria for this study were as follows: 1) patients ≥18 years old; 2) Eastern Cooperative Oncology Group performance scores ≤1; 3) no prior HCC-related treatment including resection, local ablation, systemic therapy, or TACE; 4) Child-Pugh (CP) liver function stage of A to B7; 5) aspartate aminotransferase (AST) or alanine aminotransferase (ALT) < 5 * upper limit of normal (ULN), serum creatinine <1.5 * ULN, and bilirubin level< 3 mg/dl; and 6) adequate hematologic and clotting function. The exclusion criteria were as follows: 1) comorbidity with other primary malignancies; 3) prior chemotherapy, radiotherapy, or molecular-targeted HCC therapy; 4) contraindications for embolization or sorafenib; and 5) the administration of sorafenib discontinued more than one month.

### Treatment Protocol

All the patients included in this study were treated with the conventional TACE procedure according to institutional standards, and all the TACE procedures were performed by six board-certified interventional radiologists with more than 8 years of experience. Selective or superselective catheterization of the tumor-feeding arteries was introduced with a 2.7 F microcatheter (Progreat; Terumo, Japan) after the diagnostic angiography of the celiac trunk and superior mesenteric artery with a 5 F catheter. Patients received the intra-arterial injection of an emulsion of doxorubicin (10–50 mg) and oxaliplatin (100–200 mg) in ethiodized oil (2–20 ml, Lipiodol Ultra-Fluide; Laboratoire Guerbet, Roissy Charles de Gaulle, France) followed by Gelfoam (Ailikang Inc., Hangzhou, China) particles (350–560 μm) under fluoroscopic guidance until arterial inflow was substantially reduced. After 5 min, another angiography was obtained from the common hepatic artery to verify no residual tumor enhancement. According to the “on-demand” basis in the setting of detecting new or residual tumor tissue (i.e., incomplete necrosis) on follow-up imaging, chemoembolization was repeated.

The initial administration of sorafenib (400 mg, twice daily) was within 1 week after the initial and on-demand chemoembolization. The dose of sorafenib was adjusted (400 mg/day, 400 mg every other day) for drug-related adverse events (AEs), which were based on the Common Terminology Criteria of Adverse Events (CTCAE) version 5.0. The treatment was discontinued when the patients had untreatable progression and unacceptable toxicity.

### Outcomes and Follow-Up

All the clinical and radiological data were retrieved from the electronic medical record from the two institutions. NLR was calculated as absolute neutrophil count divided by absolute lymphocyte count measured in the peripheral blood before the initial TACE treatment. PLR was calculated by division of absolute thrombocytes and lymphocytes accordingly. Baseline characteristics, including blood routine examination and biochemical analysis, were obtained 3–7 days before the initial chemoembolization and post-TACE hospitalization and every month outpatient clinical follow-up. The status of patients (alive or dead) was recorded on the medical records or inquired by phone from the family member. Multiphase Computed Tomography (CT) or Magnetic Resonance Imaging (MRI) was performed 1 week before and between 1 and 2 months after the TACE procedure. All CT scans were with 64 or more row systems and all MRI scans were 3 T unit. The evaluation of radiological response was carried out by two radiologists with abdominal imaging experience of more than 5 years. Both of them were blinded to the treatment regimen and patient information. The response of the combination treatment was classified based on the modified response evaluation criteria in solid tumors (mRECIST) (Llovet and Lencioni, 2020).

The primary outcome measurement of this study was OS. OS was defined as the duration of time from the initial TACE treatment to the date of death or the last follow-up (July 31, 2020). The second outcome measurement was PFS, which was defined as the time from the initial chemoembolization to death or radiological progression. Patients who were alive and without progression were censored at the last follow-up period.

### Statistical Analysis

Continuous variables and categorical variables were presented as median (interquartile range) and frequencies (percentages), respectively. The median value of the NLR, PLR, and aspartate transaminase (AST)/alanine transaminase (ALT) ratio was considered as the cut-off value. The cut-off values of age, AST, ALT, tumor size, number of nodules, alpha-fetoprotein (AFP), and bilirubin were based on previous studies (Song et al., 2012; Lee et al., 2019; Zhong et al., 2019). The differences of the baseline characteristics were compared between the high and low NLR/PLR groups using the Mann–Whitney *U* test or Fisher’s exact test. OS and PFS were plotted using the Kaplan–Meier method and were compared using the log-rank test. Univariate Cox’s proportional hazards regression model analysis was performed to determine the factors associated with OS and PFS. Multivariable analysis was carried out on variables that reached *p* < 0.05 at univariable analysis. Considering that NLR and PLR both take into account lymphocyte count, two separate models for NLR (model 1)/PLR (model 2) were developed for the multivariable analyses. The predictive value of NLR and PLR was also assessed by calculating the area under the curve (AUC) from receiver operating characteristic (ROC) curves. The significance level of 5% was used to determine statistical significance. All statistical analyses were performed using the SPSS (version 25.0, IBM Corp.) and R software version 3.2.2 (http://www.r-project.org).

## Results

### Patient Characteristics

A total of 314 treatment-naïve unresectable HCC patients treated with chemoembolization plus sorafenib were enrolled in this study. Of the included patients, there were 270 (86.0%) males and 44 (14.0%) females with a median age of 55 (range, 26–81) years in the entire cohort. Hepatitis B virus (HBV) (85.7%) was the predominant etiology of liver disease. The number of patients with portal vein invasion and hepatic vein invasion was 106 (33.8%) and 37 (11.8%), respectively. Nearly all patients had a CP A liver function and good performance (ECOG 0). Barcelona Clinic Liver Cancer (BCLC) stage B patients constituted 54.1% of the entire cohort. The median values of NLR and PLR were 2.42 and 100, respectively. The difference in the baseline characteristics of the two NLR/PLR groups was presented in Table 1. Compared with the patients in the high NLR group, those with low NLR had a less advanced-stage disease (BCLC stage, portal vein invasion, and extrahepatic spread), better preserved liver function (albumin-bilirubin, ALBI), and lower value of AST. Additionally, patients in the low PLR group were associated with more males, less advanced-stage disease, lower value of AFP, and lower AST/ALT ratio.

**TABLE 1:** Comparison of the clinic-laboratory data and demographic features between patients with 1) low NLR and high NLR and 2) low and high PLR.

Characteristic	Overall (*n* = 314)	NLR < 2.42 (*n* = 159)	NLR ≥ 2.42 (*n* = 155)	*p* value	PLR < 100 (*n* = 157)	PLR ≥ 100 (*n* = 157)	*p* value
Gender				0.927			0.034
Male	270 (86.0)	137 (86.2)	133 (85.8)		142 (90.5)	128 (81.5)	
Female	44 (14.0)	22 (13.8)	22 (14.2)		15 (9.5)	29 (18.45)	
Age (years)				0.431			0.366
≤55	165 (52.6)	80 (50.3)	85 (54.8)		87 (55.4)	78 (49.7)	
>55	149 (47.4)	79 (49.7)	70 (45.2)		70 (44.6)	79 (50.3)	
ECOG				0.151			1.000
0	312 (99.4)	159 (100)	153 (98.7)		156 (99.4)	156 (99.4)	
1	2 (0.6)	0 (0)	2 (1.3)		1 (0.6)	1 (0.6)	
Etiology				0.422			0.107
HBV	269 (85.7)	139 (87.4)	130 (83.9)		140 (89.2)	129 (82.2)	
Other	45 (14.3)	20 (12.6)	25 (16.1)		17 (10.8)	28 (17.8)	
Cirrhosis				0.715			0.713
	218 (69.4)	112 (70.4)	106 (68.4)		111 (70.7)	107 (68.2)	
Tumor size (cm)				0.496			0.256
≤5	175 (55.7)	92 (57.9)	83 (53.6)		93 (59.2)	82 (52.2)	
>5	139 (44.3)	67 (42.1)	72 (46.4)		64 (40.8)	75 (47.8)	
No. of nodules				0.501			1.000
<3	160 (51.0)	78 (49.1)	82 (52.9)		80 (51.0)	80 (51.0)	
≥3	154 (49.0)	81 (50.9)	73 (47.1)		77 (49.0)	77 (49.0)	
PVTT				0.001			0.042
	106 (33.8)	39 (24.5)	67 (43.2)		44 (28.0)	62 (39.5)	
Hepatic vein invasion				0.116			0.161
	37 (11.8)	14 (8.8)	23 (14.8)		14 (8.9)	23 (14.7)	
Child-Pugh class				0.980			1.000
A	310 (98.7)	157 (98.7)	153 (98.7)		155 (98.7)	155 (98.7)	
B	4 (1.3)	2 (1.3)	2 (1.3)		2 (1.3)	2 (1.3)	
BCLC stage				<0.001			0.001
B	170 (54.1)	107 (67.3)	63 (40.7)		100 (63.7)	70 (44.6)	
C	144 (45.9)	52 (32.7)	92 (59.3)		57 (36.3)	87 (55.4)	
ALBI grade				0.046			0.191
1	170 (54.1)	97 (61.0)	73 (47.1)		93 (59.2)	77 (49.0)	
2	142 (45.2)	61 (38.4)	81 (52.3)		63 (40.1)	79 (50.3)	
3	2 (0.7)	1 (0.6)	1 (0.6)		1 (0.7)	1 (0.7)	
Tumor distribution				0.282			0.905
Unilobar	210 (66.9)	111 (69.8)	99 (63.9)		106 (67.5)	104 (66.2)	
Bilobar	104 (33.1)	48 (30.2)	56 (36.1)		51 (32.5)	53 (33.8)	
Extrahepatic spread (PVTT excluded)	32 (10.2)	8 (5.0)	24 (15.5)	0.003	10 (6.4)	22 (14.0)	0.039
AFP (ng/dl)				0.070			0.024
≤200	167 (53.2)	93 (58.5)	74 (47.7)		94 (59.9)	73 (45.5)	
>200	147 (46.8)	66 (41.5)	81 (52.3)		63 (40.1)	84 (54.5)	
AST (U/L)				0.003			0.054
≤40	144 (45.9)	86 (54.1)	58 (37.4)		81 (51.6)	63 (40.1)	
>40	170 (54.1)	73 (45.9)	97 (62.6)		76 (48.4)	94 (59.9)	
ALT (U/L)				0.909			0.087
≤40	180 (57.3)	92 (57.9)	88 (56.8)		82 (52.2)	98 (62.4)	
>40	134 (42.7)	67 (42.1)	67 (42.2)		75 (47.7)	59 (37.6)	
Albumin (g/L)				0.595			0.894
≤35	74 (23.6)	35 (22.0)	39 (25.2)		38 (24.2)	36 (22.9)	
>35	240 (76.4)	124 (78.0)	116 (74.8)		119 (75.8)	121 (77.1)	
TBIL (umol/L)				0.107			0.227
≤17.1	242 (77.1)	129 (81.1)	113 (72.9)		116 (73.9)	126 (80.2)	
>17.1	72 (22.9)	30 (18.9)	42 (27.1)		41 (26.1)	31 (19.8)	
AST/ALT				0.178			0.005
≤1.18	156 (49.7)	85 (53.5)	71 (45.8)		91 (58.0)	65 (41.4)	
>1.18	158 (50.3)	74 (46.5)	84 (54.2)		66 (42.0)	92 (58.6)	

AFP, alpha-fetoprotein; ALBI, albumin-bilirubin; ALT, alanine transaminase; AST, aspartate transaminase; BCLC, Barcelona Clinic Liver Cancer; ECOG, Eastern Cooperative Oncology Group; HBV, hepatitis B virus; NLR, neutrophil-to-lymphocyte ratio; PLR, platelet-to-lymphocyte ratio; PVTT, portal vein tumor thrombus; TBIL, total bilirubin.

### Outcomes

**FIGURE 1:**
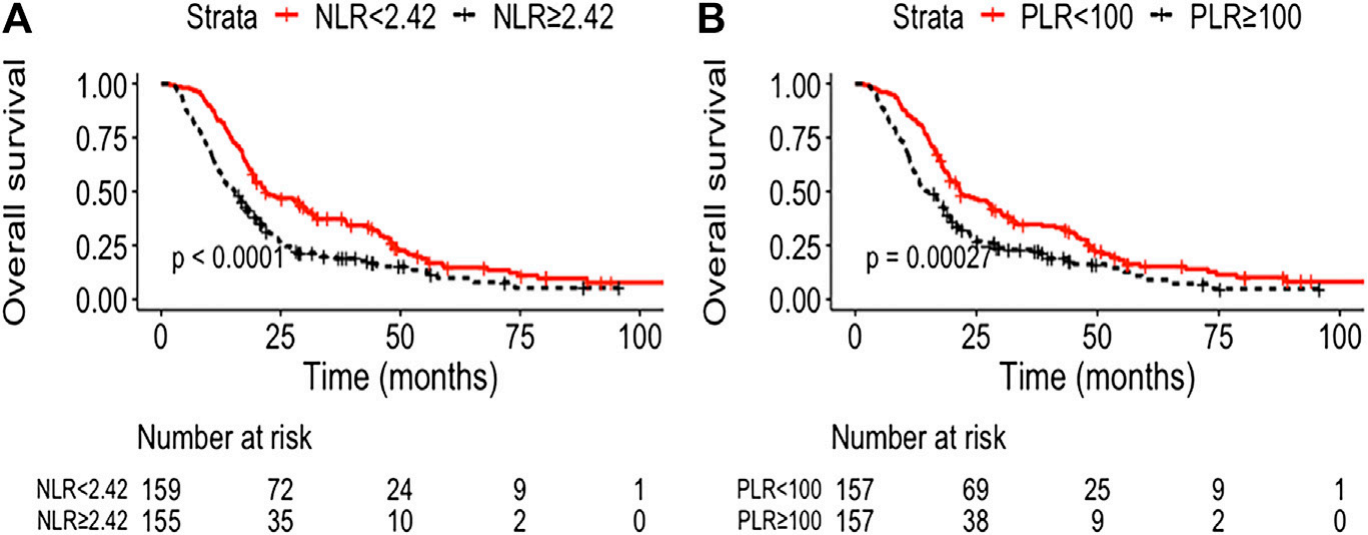
Kaplan–Meier curves of different group analyses of overall survival (OS) according to patients’ NLR and PLR level. **(A)** Patients with low NLR vs. high NLR at baseline; **(B)** patients with low PLR vs. high PLR at baseline.

**FIGURE 2:**
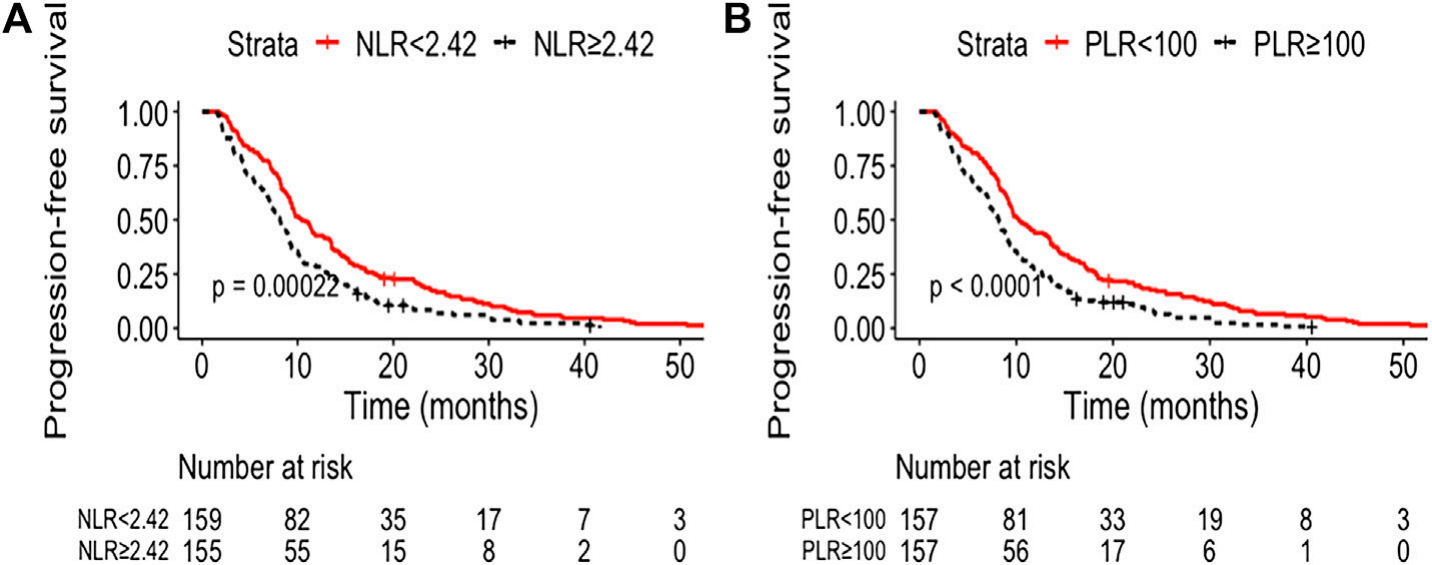
Kaplan–Meier curves of different group analyses of progression-free survival (PFS) according to patients’ NLR and PLR level. **(A)** Patients with low NLR vs. high NLR at baseline; **(B)** patients with low PLR vs. high PLR at baseline.

**FIGURE 3:**
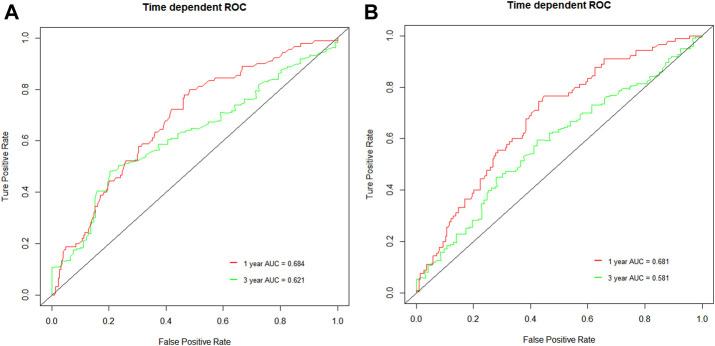
ROC curves of the NLR and PLR in unresectable HCC patients. **(A)** 1-year and 3-year ROC curve of NLR; **(B)** 1-year and 3-year ROC curve of PLR.

The median follow-up was 21.0 months (95% CI, 19.7–22.4). The median OS and PFS of the entire cohort were 18.7 months (95% CI: 16.8–20.6) and 9.1 months (95% CI: 8.5–9.8), respectively. The low NLR and PLR group showed improved OS compared with the high NLR and PLR group [21.8 months (95% CI: 15.2–28.5) vs. 15.4 months (95% CI: 12.4–18.3), *p* < 0.0001; 21.6 months (95% CI: 15.8–27.5) vs. 14.9 months (95% CI: 11.9–17.8), *p* = 0.00027, respectively] (Figure 1). The 1- and 3-year OS rate of the low and high NLR/PLR group was 83.0% vs. 59.4% and 36.6% vs. 18.9% and 83.4% vs. 59.2% and 34.0% vs. 22.2%, respectively. In addition, the low NLR and PLR group also provided a longer PFS than the high NLR and PLR group [10.4 months (95% CI: 8.9–12.0) vs. 8.1 months (95% CI: 7.1–9.2), *p* = 0.00022; 10.3 months (95% CI: 8.6–11.9) vs. 8.2 months (95% CI: 7.2–9.2), *p* < 0.0001, respectively] (Figure 2). The 1-year PFS rate of the low and high NLR/PLR group was 42.8% vs. 28.4% and 43.9% vs. 27.4%, respectively. The 1-year AUC of NLR and PLR was 0.684 and 0.681, respectively. The cut-off values of NLR and PLR correspond to sensitivity values of 54.8% and 53.9%, and specificity values of 45.2% and 42.8%, respectively (Figure 3A). The 3-year AUC of NLR and PLR was 0.621 and 0.581, respectively. The NLR and PLR correspond to sensitivity values of 45.5% and 46.1%, and specificity values of 42.8% and 55.4%, respectively (Figure 3B).

### Prognostic Factors

In the univariate survival analysis, tumor size, portal vein invasion, extrahepatic spread, ALBI, AFP, AST, albumin, NLR, PLR and AST/ALT ratio were associated with OS (*p* < 0.05 for all) (Table 2). Multivariable Cox regression analysis indicated that in model 1, high NLR, tumor size >5 cm, portal vein invasion, and extrahepatic spread were independent factors associated with poor OS, whereas model 2 showed high PLR, portal vein invasion, AST >40 U/L, and extrahepatic spread as prognostic for poorer OS in patients receiving chemoembolization plus sorafenib (Table 3).

**TABLE 2:** Univariate analysis of risk factors associated with overall survival.

Characteristic		HR	95% CI	*p* value
Gender	F/M	0.883	0.618–1.261	0.493
Age (years)	>55/≤55	0.813	0.636–1.040	0.099
ECOG	1/0	0.370	0.052–2.655	0.323
Etiology	HBV/others	0.917	0.652–1.290	0.619
Cirrhosis	Yes/no	0.928	0.713–1.209	0.582
Tumor size (cm)	>5/≤5	1.750	1.362–2.249	<0.001
No. of nodules	≥3/<3	1.227	0.961–1.567	0.101
PVTT	Yes/no	1.864	1.429–2.432	<0.001
Hepatic vein invasion	Yes/no	1.283	0.868–1.895	0.211
Child-Pugh class	B/A	1.597	0.593–4.297	0.354
ALBI grade	3/2/1	1.389	1.095–1.762	0.007
Tumor distribution	Bilobar/unilobar	1.146	0.887–1.479	0.297
Extrahepatic spread	Yes/no	2.461	1.673–3.621	<0.001
AFP (ng/dl)	>200/≤200	1.418	1.107–1.817	0.006
AST (U/L)	>40/≤40	1.479	1.155–1.894	0.002
ALT (U/L)	>40/≤40	1.129	0.883–1.443	0.334
Albumin (g/L)	>35/≤35	0.698	0.526–0.926	0.013
TBIL (umol/L)	>17.1/≤17.1	1.089	0.754–1.573	0.648
NLR	≥2.42/<2.42	1.647	1.287–2.109	<0.001
PLR	≥100/<100	1.576	1.231–2.018	<0.001
AST/ALT	>1.18/≤1.18	1.328	1.037–1.701	0.025

AFP, alpha-fetoprotein; ALBI, albumin-bilirubin; ALT, alanine transaminase; AST, aspartate transaminase; ECOG, Eastern Cooperative Oncology Group; HBV, hepatitis B virus; NLR, neutrophil-to-lymphocyte ratio; PLR, platelet-to-lymphocyte ratio; PVTT, portal vein tumor thrombus; TBIL, total bilirubin; HR, hazard ratio; CI, confidence interval.

**TABLE 3:** Multivariate Cox proportional hazards regression analysis of NLR and PLR with overall survival.

Characteristic		B	SE B	Wald	HR	95% CI	*p* value
NLR							
Tumor size (cm)	>5/≤5	0.344	0.136	6.434	1.410	1.081–1.840	0.011
PVTT	Yes/no	0.466	0.141	10.950	1.593	1.209–2.099	0.001
Extrahepatic spread	Yes/no	0.708	0.202	12.229	2.029	1.365–3.017	<0.001
NLR	≥2.42/<2.42	0.305	0.131	5.4333	1.356	1.050–1.753	0.020
PLR							
PVTT	Yes/no	0.487	0.140	12.133	1.627	1.237–2.139	<0.001
Extrahepatic spread	Yes/no	0.915	0.197	21.544	2.497	1.697–3.674	<0.001
AST	>40/≤40	0.298	0.129	5.368	1.348	1.047–1.735	0.021
PLR	≥100/<100	0.366	0.128	8.120	1.442	1.121–1.855	0.004

AST, aspartate transaminase; NLR, neutrophil-to-lymphocyte ratio; PLR, platelet-to-lymphocyte ratio; PVTT, portal vein tumor thrombus; SE, standard error; CI, confidence interval; HR, hazard ratio.

The univariate analysis identified tumor size, portal vein invasion, hepatic vein invasion, extrahepatic spread, AFP, AST, albumin, NLR, and PLR as potential prognostic factors for PFS (Table 4). The multivariable analysis using model 1 identified tumor size >5 cm, AST >40 U/L, extrahepatic spread, and high NLR as prognostic for shorter PFS, and the model showed similar results with tumor size >5 cm, AST >40 U/L, and extrahepatic spread as prognostic for poorer PFS apart from high PLR (Table 5).

**TABLE 4:** Univariate analysis of risk factors associated with progression-free survival.

Characteristic		HR	95% CI	*p* value
Gender	F/M	1.030	0.743–1.428	0.858
Age (years)	>55/≤55	0.911	0.728–1.140	0.415
ECOG	1/0	0.562	0.139–2.273	0.419
Etiology	HBV/others	1.030	0.745–1.423	0.859
Cirrhosis	Yes/no	0.935	0.734–1.193	0.591
Tumor size (cm)	>5/≤5	1.588	1.262–1.997	<0.001
No. of nodules	≥3/<3	1.020	0.813–1.280	0.865
PVTT	Yes/no	1.576	1.234–2.014	<0.001
Hepatic vein invasion	Yes/no	1.477	1.043–2.093	0.028
Child-Pugh class	B/A	1.313	0.488–3.529	0.589
ALBI grade	3/2/1	1.169	0.941–1.452	0.158
Tumor distribution	Bilobar/unilobar	0.915	0.719–1.165	0.472
Extrahepatic spread	Yes/no	2.091	1.442–3.031	<0.001
AFP (ng/dl)	>200/≤200	1.321	1.053–1.656	0.016
AST (U/L)	>40/≤40	1.545	1.229–1.942	<0.001
ALT (U/L)	>40/≤40	1.033	0.822–1.297	0.783
Albumin (g/L)	>35/≤35	0.751	0.575–0.981	0.036
TBIL (umol/L)	>17.1/≤17.1	1.079	0.778–1.499	0.648
NLR	≥2.42/<2.42	1.533	1.220–1.926	<0.001
PLR	≥100/<100	1.599	1.270–2.013	<0.001
AST/ALT	>1.18/≤1.18	1.424	1.135–1.787	0.002

AFP, alpha-fetoprotein; ALBI, albumin-bilirubin; ALT, alanine transaminase; AST, aspartate transaminase; ECOG, Eastern Cooperative Oncology Group; HBV, hepatitis B virus; NLR, neutrophil-to-lymphocyte ratio; PLR, platelet-to-lymphocyte ratio; PVTT, portal vein tumor thrombus; TBIL, total bilirubin; HR, hazard ratio; CI, confidence interval.

**TABLE 5:** Multivariate Cox proportional hazards regression analysis of NLR and PLR with progression-free survival.

Characteristic		B	SE B	Wald	HR	95% CI	*p* value
NLR							
Tumor size (cm)	>5/≤5	0.303	0.123	6.091	1.354	1.064–1.722	0.014
AST (U/L)	>40/≤40	0.378	0.121	9.712	1.459	1.151–1.850	0.002
Extrahepatic spread	Yes/no	0.378	0.121	9.712	1.459	1.151–1.850	0.002
NLR	≥2.42/<2.42	0.246	0.123	4.013	1.278	1.005–1.626	0.045
PLR							
Tumor size (cm)	>5/≤5	0.271	0.123	4.865	1.312	1.031–1.669	0.027
AST (U/L)	>40/≤40	0.390	0.120	10.643	1.477	1.169–1.868	0.001
Extrahepatic spread	Yes/no	0.735	0.193	14.500	2.086	1.429–3.045	<0.001
PLR	≥100/<100	0.345	0.122	8.063	1.412	1.113–1.792	0.005

AST, aspartate transaminase; NLR, neutrophil-to-lymphocyte ratio; PLR, platelet-to-lymphocyte ratio; SE, standard error; CI, confidence interval; HR, hazard ratio.

## Discussion

This study demonstrated that hematological parameters (NLR or PLR) at baseline could predict outcomes of unresectable HCC patients undergoing the combination therapy of chemoembolization plus sorafenib. Patients with NLR <2.42 or PLR <100 had an improved OS or PFS than that in the high NLR or PLR group. Two models, which separately included NLR and PLR, both showed NLR and PLR were strong prognostic factors, indicating that NLR or PLR was useful to differentiate target patients and stratify risk.

More recently, the first-ever positive TACTIS trial showed the superiority of TACE in combination of sorafenib over TACE alone in terms of clinical outcomes, including PFS (25.2 vs. 13.5 months; HR = 0.59; 95% CI: 0.41–0.87; *p* = 0.006) and 1- and 2-year survival rates (96.2% vs. 82.7%; 77.2% vs. 64.6%, respectively), indicating that unresectable HCC patients could benefit from the combination therapy (Kudo et al., 2020). The better outcomes provided by this trial may due to the normalization of feeding arteries, leading to enhancement of TACE efficacy through dense accumulation of embolization agents (i.e., lipiodol mixed with chemotherapeutic drugs followed by Gelfoam). The liver function preservation caused by less TACE repetition and a much longer duration of sorafenib administration could be the other possible explanations (Kudo et al., 2020). However, considering that the biological heterogeneity of unresectable HCC and the tumor microenvironment could hamper treatment efficacy, resulting in heterogeneous prognosis in individuals, new biomarkers are warranted in order to evaluate the impact of the individual immune system activity on tumor progression and susceptibility to the combination therapy of chemoembolization plus sorafenib (Schobert et al., 2020).

As measured in peripheral blood samples, NLR and PLR were considered as indirect markers of systemic inflammatory response and have been evaluated as predictors of recurrence and survival in various malignancies (Templeton et al., 2014; Zheng et al., 2017b). Several meta-analysis studies suggested that high NLR and PLR are associated with an adverse OS in HCC patients undergoing liver transplantation or hepatectomy (Lin et al., 2018; Wang et al., 2018). More recently, a meta-analysis including 5280 HCC patients treated by TACE reported that evaluated NLR and PLR at baseline were significantly correlated with poor OS (HR: 1.81, 95% CI: 1.66–1.97, *p* < 0.00001; HR: 1.56, 95% CI: 1.13–2.16, *p* = 0.007, respectively) (Li et al., 2020). In addition, another meta-analysis indicated that HCC patients with lower NLR at baseline could have a better response to sorafenib than those with higher pretreatment NLR (HR = 1.76, 95% CI: 1.44–2.15, *p* < 0.00001) (Liu et al., 2019). As shown in the present study, lower NLR and PLR were significant predictive factors for better survival in the unresectable HCC patients treated with TACE plus sorafenib (HR = 1.36, 95% CI: 1.05–1.75, *p* = 0.02; HR = 1.44, 95% CI: 1.12–1.86, *p* = 0.004, respectively). The similar prognostic values of NLR and PLR in patients undergoing the combination therapy may result from the following reasons. First, given HBV induces chronic inflammation and immune modulation, patients with either a higher adaptive immune infiltrate (lymphocytes) or lower innate immune infiltrate (neutrophils or platelet) may have a better response to sorafenib. Additionally, previous evidence showed that the immune system and tumor microenvironment could be affected by sorafenib with enhancing T-cell activation and blocking T-cell regulatory function (Bruix et al., 2017). Second, although TACE-induced tumor hypoxia may have an impact on immune cell activity, TACE has the potential to affect the immune system in a positive way by exposing tumor antigens to the immune system (Xue et al., 2015). A recent experimental study illustrated that the specialized subset of T helper lymphocytes (Th17) and its signature cytokine IL-17 were increased after the embolization treatment (Avritscher et al., 2020). Tampaki et al. reported the sTIM-3 level in plasma significantly increased after TACE due to the upregulation, whereas patients with better response had higher posttreatment values (Tampaki et al., 2020).

In the present study, the OS and PFS curve showed a cut-off value of median NLR and PLR of 2.42 and 100, which significantly patients’ OS and PFS stratified based on NLR and PLR (*p* < 0.001 for all). A recent meta-analysis indicated that 3 was the minimum cut-off value for NLR to play a prognostic value (Liu et al., 2019). However, different ethnic populations and the heterogeneity of the enrolled studies had a greater impact on the consistency of the results. Another study conducted by Wang et al. suggested that baseline NLR >2.4 was an independent prognostic factor of poor OS, which was similar to this study. For the cut-off value of PLR, it was also similar to a previous study which illustrated that lower preoperative PLR (≤100) can predict longer disease-free survival (DFS) and OS for HCC patients undergoing TACE plus radiofrequency ablation (RFA) (Long et al., 2020). Moreover, more patients with less advanced stage were shown in both low NLR and PLR groups in this study; previous evidence also showed the incidence of high pretreatment NLR had a significant association with the presence of portal vein invasion (Li et al., 2020).

It should be noted that there are some limitations in this study. First, select bias may exist in this study due to the retrospective nature. Second, the target population of this study included patients with portal vein invasion, which was a relative contraindication of TACE. However, previous studies showed that advanced HCC patients treated with chemoembolization plus sorafenib could have favorable outcomes compared to those treated with sorafenib monotherapy (Zhang et al., 2020a). Last but not least, this study did not investigate the prognostic value and the other inflammatory and immune biomarkers. Well-designed prospective studies are warranted to evaluate the meaning of the other biomarkers and also conduct a clinically meaningful cut-off value.

In conclusion, this study reported the feasibility and validated two prognostic biomarkers (NLR or PLR) for unresectable HCC patients following the combination therapy of chemoembolization plus sorafenib and indicated that unresectable HCC patients with lower NLR/PLR may have a more favorable outcome than those with high ones. These biomarkers could be easily implemented in routine practice, which may be explored as a paradigm for physicians in the treatment decision.

## Data Availability

The original contributions presented in the study are included in the article/Supplementary Material; further inquiries can be directed to the corresponding author.

## Abbreviations

AEs, adverse events; ALBI, albumin-bilirubin; AST, aspartate aminotransferase; BCLC, Barcelona Clinic Liver Cancer; CI, confidence interval; CT, computed tomography; EASL, European Association for the Study of the Liver; ECOG, Eastern Cooperative Oncology Group; HCC, hepatocellular carcinoma; IRBs, Institutional Review Boards; mRECIST, modified response evaluation criteria in solid tumors; MRI, magnetic resonance imaging; OS, overall survival; PFS, progression-free survival; TACE, transarterial chemoembolization; VEGF, vascular endothelial growth factor.l.

## References

[B1] Avritscher R. Jo N. Polak U. Cortes A. C. Nishiofuku H. Odisio B. C. (2020). Hepatic arterial bland embolization increases Th17 cell infiltration in a syngeneic rat model of hepatocellular carcinoma. Cardiovasc. Interv. Radiol. 43 (2), 311–321. 10.1007/s00270-019-02343-1 31591689

[B2] Bruix J. Cheng A.-L. Meinhardt G. Nakajima K. De Sanctis Y. Llovet J. (2017). Prognostic factors and predictors of sorafenib benefit in patients with hepatocellular carcinoma: analysis of two phase III studies. J. Hepatol. 67 (5), 999–1008. 10.1016/j.jhep.2017.06.026 28687477

[B3] Cheng A.-L. Kang Y.-K. Chen Z. Tsao C.-J. Qin S. Kim J. S. (2009). Efficacy and safety of sorafenib in patients in the Asia-Pacific region with advanced hepatocellular carcinoma: a phase III randomised, double-blind, placebo-controlled trial. Lancet Oncol. 10 (1), 25–34. 10.1016/s1470-2045(08)70285-7 19095497

[B4] Chon Y. E. Park H. Hyun H. K. Ha Y. Kim M. N. Kim B. K. (2019). Development of a new nomogram including neutrophil-to-lymphocyte ratio to predict survival in patients with hepatocellular carcinoma undergoing transarterial chemoembolization. Cancers 11 (4), 509. 10.3390/cancers11040509 PMC6520830 30974843

[B5] European Association for the Study of the Liver (2018). EASL clinical practice guidelines: management of hepatocellular carcinoma. J. Hepatol. 69 (1), 182–236. 10.1016/j.jhep.2018.03.019 29628281

[B6] Forner A. Reig M. Bruix J. (2018). Hepatocellular carcinoma. Lancet 391 (10127), 1301–1314. 10.1016/S0140-6736(18)30010-2 29307467

[B7] He C. Zhang Y. Cai Z. Lin X. (2019). The prognostic and predictive value of the combination of the neutrophil-to-lymphocyte ratio and the platelet-to-lymphocyte ratio in patients with hepatocellular carcinoma who receive transarterial chemoembolization therapy. Cancer Manag. Res. 11, 1391–1400. 10.2147/cmar.s190545 30863150 PMC6388940

[B8] Hong Y. M. Yoon K. T. Hwang T. H. Heo J. Woo H. Y. Cho M. (2019). Changes in the neutrophil-to-lymphocyte ratio predict the prognosis of patients with advanced hepatocellular carcinoma treated with sorafenib. Eur. J. Gastroenterol. Hepatol. 31 (10), 1250–1255. 10.1097/meg.0000000000001405 30925530

[B9] Kim Y. S. Shin S. W. (2019). Hepatocellular carcinoma. N. Engl. J. Med. 381 (1), e2. 10.1056/NEJMc1906565 31269385

[B10] Kudo M. Imanaka K. Chida N. Nakachi K. Tak W.-Y. Takayama T. (2011). Phase III study of sorafenib after transarterial chemoembolisation in Japanese and Korean patients with unresectable hepatocellular carcinoma. Eur. J. Cancer 47 (14), 2117–2127. 10.1016/j.ejca.2011.05.007 21664811

[B11] Kudo M. (2018). Proposal of primary endpoints for TACE combination trials with systemic therapy: lessons learned from 5 negative trials and the positive TACTICS trial. Liver cancer 7 (3), 225–234. 10.1159/000492535 30319982 PMC6167729

[B12] Kudo M. Ueshima K. Ikeda M. Torimura T. Tanabe N. Aikata H. (2020). Randomised, multicentre prospective trial of transarterial chemoembolisation (TACE) plus sorafenib as compared with TACE alone in patients with hepatocellular carcinoma: TACTICS trial. Gut 69 (8), 1492–1501. 10.1136/gutjnl-2019-318934 31801872 PMC7398460

[B13] Lee I. C. Hung Y.-W. Liu C.-A. Lee R.-C. Su C.-W. Huo T.-I. (2019). A new ALBI-based model to predict survival after transarterial chemoembolization for BCLC stage B hepatocellular carcinoma. Liver Int. Off. J. Int. Assoc. Study Liver 39 (9), 1704–1712. 10.1111/liv.14194 31319016

[B14] Lencioni R. Llovet J. M. Han G. Tak W. Y. Yang J. Guglielmi A. (2016). Sorafenib or placebo plus TACE with doxorubicin-eluting beads for intermediate stage HCC: the SPACE trial. J. Hepatol. 64 (5), 1090–1098. 10.1016/j.jhep.2016.01.012 26809111

[B15] Li S. Feng X. Cao G. Wang Q. Wang L. (2020). Prognostic significance of inflammatory indices in hepatocellular carcinoma treated with transarterial chemoembolization: a systematic review and meta-analysis. PloS One 15 (3), e0230879. 10.1371/journal.pone.0230879 32214401 PMC7098645

[B16] Lin W. F. Zhong M. F. Zhang Y. R. Wang H. Zhao H. T. Cheng B. B. (2018). Prognostic role of platelet-to-lymphocyte ratio in hepatocellular carcinoma with different BCLC stages: a systematic review and meta-analysis. Gastroenterol. Res. Pract. 2018, 5670949. 10.1155/2018/5670949 30158964 PMC6109515

[B17] Liu L. Gong Y. Zhang Q. Cai P. Feng L. (2019). Prognostic roles of blood inflammatory markers in hepatocellular carcinoma patients taking sorafenib. A systematic review and meta-analysis. Front. Oncol. 9, 1557. 10.3389/fonc.2019.01557 32064238 PMC7000550

[B18] Llovet J. M. Lencioni R. (2020). mRECIST for HCC: performance and novel refinements. J. Hepatol. 72 (2), 288–306. 10.1016/j.jhep.2019.09.026 31954493 PMC12452114

[B19] Long J. Wang H. Zhao P. Sheng S. P. Qin-Sheng S. Long M. (2020). Transarterial chemoembolization combined with radiofrequency ablation for solitary large hepatocellular carcinoma ranging from 5 to 7 cm: an 8-year prospective study. Abdom. Radiol. (NY) 45 (9), 2736–2747. 10.1007/s00261-020-02612-5 32533245

[B20] Meyer T. Fox R. Ma Y. T. Ross P. J. James M. W. Sturgess R. (2017). Sorafenib in combination with transarterial chemoembolisation in patients with unresectable hepatocellular carcinoma (TACE 2): a randomised placebo-controlled, double-blind, phase 3 trial. Lancet Gastroenterol. Hepatol. 2 (8), 565–575. 10.1016/s2468-1253(17)30156-5 28648803

[B21] Park J. W. Chen M. Colombo M. Roberts L. R. Schwartz M. Chen P. J. (2015). Global patterns of hepatocellular carcinoma management from diagnosis to death: the BRIDGE Study. Liver Int. 35 (9), 2155–2166. 10.1111/liv.12818 25752327 PMC4691343

[B22] Pawlik T. M. Reyes D. K. Cosgrove D. Kamel I. R. Bhagat N. Geschwind J.-F. H. (2011). Phase II trial of sorafenib combined with concurrent transarterial chemoembolization with drug-eluting beads for hepatocellular carcinoma. J. Clin. Oncol. Off. J. Am. Soc. Clin. Oncol. 29 (30), 3960–3967. 10.1200/jco.2011.37.1021 PMC4829081 21911714

[B23] Schobert I. T. Savic L. J. Chapiro J. Bousabarah K. Chen E. Laage-Gaupp F. (2020). Neutrophil-to-lymphocyte and platelet-to-lymphocyte ratios as predictors of tumor response in hepatocellular carcinoma after DEB-TACE. Eur. Radiol. 30 (10), 5663–5673. 10.1007/s00330-020-06931-5 32424595 PMC7483919

[B24] Song M. J. Chun H. J. Song D. S. Kim H. Y. Yoo S. H. Park C.-H. (2012). Comparative study between doxorubicin-eluting beads and conventional transarterial chemoembolization for treatment of hepatocellular carcinoma. J. Hepatol. 57 (6), 1244–1250. 10.1016/j.jhep.2012.07.017 22824821

[B25] Tampaki M. Ionas E. Hadziyannis E. Deutsch M. Malagari K. Koskinas J. (2020). Association of TIM-3 with BCLC stage, serum PD-L1 detection, and response to transarterial chemoembolization in patients with hepatocellular carcinoma. Cancers 12 (1), 212. 10.3390/cancers12010212 PMC7016746 31952209

[B26] Templeton A. J. McNamara M. G. Seruga B. Vera-Badillo F. E. Aneja P. Ocana A. (2014). Prognostic role of neutrophil-to-lymphocyte ratio in solid tumors: a systematic review and meta-analysis. J. Natl. Cancer Inst. 106 (6), dju124. 10.1093/jnci/dju124 24875653

[B27] Wang B. Xu H. Gao Z. Q. Ning H. F. Sun Y. Q. Cao G. W. (2008). Increased expression of vascular endothelial growth factor in hepatocellular carcinoma after transcatheter arterial chemoembolization. Acta Radiol. 49 (5), 523–529. 10.1080/02841850801958890 18568538

[B28] Wang C. Wang M. Zhang X. Zhao S. Hu J. Han G. (2020). The neutrophil-to-lymphocyte ratio is a predictive factor for the survival of patients with hepatocellular carcinoma undergoing transarterial chemoembolization. Ann. Transl. Med. 8 (8), 541. 10.21037/atm.2020.02.113 32411764 PMC7214899

[B29] Wang Y. Peng C. Cheng Z. Wang X. Wu L. Li J. (2018). The prognostic significance of preoperative neutrophil-lymphocyte ratio in patients with hepatocellular carcinoma receiving hepatectomy: a systematic review and meta-analysis. Int. J. Surg. 55, 73–80. 10.1016/j.ijsu.2018.05.022 29787804

[B30] Xue T.-C. Jia Q.-A. Ge N.-L. Chen Y. Zhang B.-H. Ye S.-L. (2015). Imbalance in systemic inflammation and immune response following transarterial chemoembolization potentially increases metastatic risk in huge hepatocellular carcinoma. Tumour Biol. J. Int. Soc. Oncodevelopmental Biol. Med. 36 (11), 8797–8803. 10.1007/s13277-015-3632-7 26058874

[B31] Zhang L. Sun J. H. Hou Z. H. Zhong B. Y. Yang M. J. Zhou G. H. (2020a). Prognosis nomogram for hepatocellular carcinoma patients with portal vein invasion undergoing transarterial chemoembolization plus sorafenib treatment: a retrospective multicentre study. Cardiovasc. Intervent Radiol. 44, 63–72. 10.1007/s00270-020-02579-2 32965582

[B32] Zhang L. Xia W. Yan Z.-P. Sun J.-H. Zhong B.-Y. Hou Z.-H. (2020b). Deep learning predicts overall survival of patients with unresectable hepatocellular carcinoma treated by transarterial chemoembolization plus sorafenib. Front. Oncol. 10, 593292. 10.3389/fonc.2020.593292 33102242 PMC7556271

[B33] Zheng J. Cai J. Li H. Zeng K. He L. Fu H. (2017a). Neutrophil to lymphocyte ratio and platelet to lymphocyte ratio as prognostic predictors for hepatocellular carcinoma patients with various treatments: a meta-analysis and systematic review. Cell Physiol. Biochem. Int. J. Exp. Cell. Physiol. Biochem. Pharmacol. 44 (3), 967–981. 10.1159/000485396 29179180

[B34] Zheng J. Seier K. Gonen M. Balachandran V. P. Kingham T. P. D'Angelica M. I. (2017b). Utility of serum inflammatory markers for predicting microvascular invasion and survival for patients with hepatocellular carcinoma. Ann. Surg. Oncol. 24 (12), 3706–3714. 10.1245/s10434-017-6060-7 28840521 PMC8457436

[B35] Zhong B.-Y. Ni C.-F. Ji J.-S. Yin G.-W. Chen L. Zhu H.-D. (2019). Nomogram and artificial neural network for prognostic performance on the albumin-bilirubin grade for hepatocellular carcinoma undergoing transarterial chemoembolization. J. Vasc. Interv. Radiol. 30 (3), 330–338. 10.1016/j.jvir.2018.08.026 30819473

