# Corrigendum: Neutrophil-to-Lymphocyte and Platelet-to-Lymphocyte Ratios as Predictors of Outcomes in Patients With Unresectable Hepatocellular Carcinoma Undergoing Transarterial Chemoembolization Plus Sorafenib

**DOI:** 10.3389/fmolb.2021.727969

**Published:** 2021-08-30

**Authors:** Lei Zhang, Zhi-Ping Yan, Zhong-Heng Hou, Peng Huang, Min-Jie Yang, Shuai Zhang, Shen Zhang, Shao-Hua Zhang, Xiao-Li Zhu, Cai-Fang Ni, Qiang Li

**Affiliations:** ^1^ Department of Interventional Radiology, The First Affiliated Hospital of Soochow University, Suzhou, China; ^2^ Department of Interventional Radiology, Zhongshan Hospital, Fudan University, Shanghai, China; ^3^ Shanghai Institution of Medical Imaging, Shanghai, China; ^4^ National Clinical Research Center for Interventional Medicine, Shanghai, China; ^5^ Institute of Urology, The Affiliated Luohu Hospital of Shenzhen University, Shenzhen, China; ^6^ Department of Radiology, The Affiliated People’s Hospital of Ningbo University, Ningbo, China

**Keywords:** TACE, sorafenib, HCC, platelet count, neutrophils, lymphocytes

In the published article, there was an error regarding the affiliations for Lei Zhang and Cai-Fang Ni. One of the affiliations was added in error. This has now been removed.

The authors apologize for this error and state that this does not change the scientific conclusions of the article in any way. The original article has been updated.

